# Guideline Daily Amounts Versus Nutri-Score Labeling: Perceptions of Greek Consumers About Front-of-Pack Label

**DOI:** 10.7759/cureus.32198

**Published:** 2022-12-05

**Authors:** Lamprini Kontopoulou, Georgios E Karpetas, Ourania S Kotsiou, Evangelos C Fradelos, Ioanna V Papathanasiou, Foteini Malli, Dimitrios Papagiannis, Dimitrios C Mantzaris, Chantal Julia, Serge Hercberg, Pilar Galan, Morgane Fialon, Konstantinos I Gourgoulianis

**Affiliations:** 1 Faculty of Nursing, Respiratory Disorders Lab, University of Thessaly, Larissa, GRC; 2 Faculty of Medicine, Medical Informatics Lab, University of Thessaly, Larissa, GRC; 3 Faculty of Nursing, University of Thessaly, Larissa, GRC; 4 Faculty of Nursing, Community Nursing Lab, University of Thessaly, Larissa, GRC; 5 Nutritional Epidemiology Research Team, Université Sorbonne Paris Nord, Paris, FRA; 6 Public Health Department, Université Sorbonne Paris Nord, Paris, FRA; 7 Faculty of Medicine, Pulmonology, University General Hospital of Larissa, Larissa, GRC; 8 Faculty of Medicine, Pulmonology, University of Thessaly, Larissa, GRC

**Keywords:** behavior greek consumers, promote health, front-of-pack nutrition label, food policies, nutritional labelling

## Abstract

Nutrition information is becoming more urgent than ever so that consumers can make informed choices when buying food. This study aimed to investigate consumers' perceptions and behavior of the front-of-pack label (FOPL), between two specific labeling systems, the Nutri-Score and the Guideline Daily Amounts (GDA). This is a post hoc analysis of data from a previous, more extensive cross-sectional study conducted from April 2021 to June 2021. A total of 510 participants were included in the study, of whom 49.6% were women. Participants' perceptions were assessed in nine questions on a five-point Likert scale (1=strongly disagree, 5=strongly agree). Multivariate variance analysis (MANOVA) was performed to test the effects of the label GDA vs. Nutri-Score on the overall positive and negative perceptions. We found that the Nutri-Score label was significantly more understandable (p=0.003), clear, visible, and preferable (p<0.001) than the GDA label, which required more time to be understood. The findings indicate that the interpretive label, Nutri-Score, was superior to the non-interpretive label GDA, in terms of consumers’ perception, more visibility, less time-consuming, and reliability. The FOPL can improve the ability of consumers in Greece to understand the healthfulness of food products.

## Introduction

Nutrition information on food composition is given to consumers mainly through the nutrition declaration, which is mandatory in many countries, including the European Union (EU) [1]. According to EU legislation, prepackaged foods must bear the so-called nutrition label, which is called "nutrition declaration" to inform consumers about both their energy content and their nutritional composition [2]. The information provided through the nutrition declaration is usually located on the back or side of the package which is not easily discernible to consumers [1]. Today, with the abundance of food available, consumers are faced with the challenge of understanding exactly what they buy and what they consume. Nutrition information is becoming more urgent than ever so that consumers can make informed choices when buying food [3,4]. The nutrition label on the front of the package has been specifically recognized in packaged foods as an easy, complementary way to allow consumers to make healthier food choices [2,5,6].

From the historical perspective, in the late 1980s, the introduction and use of front-of-pack labeling began by both governmental and non-governmental organizations [7,8]. This information on the front of the food packaging is complementary and aims to understand the nutritional value of food better. This type of labeling is commonly referred to as front-of-pack label (FOPL) or front of pack (FOP) or front-of-pack nutritional labeling (FOPNL) [9]. The purpose of the nutrition label on the front of the package is complementary and is not intended to replace the nutritional information on the back or side of the packaging [5] by providing visible dietary guidelines that can affect consumer knowledge as well as food reformulation [10]. FOPL is used in more than 30 countries worldwide, but only in a few of them, it is considered mandatory [11].

According to Rebecca Kanter and her colleagues (2018) [7], in 1989, the first "Keyhole logo" was created in the Nordic European countries, followed by the Netherlands, which in 2006 introduced the "Choices logo". In 2006, the European Food and Beverage Industry Association developed and introduced the scheme called the "Guideline Daily Amounts" (GDA) which later was renamed the "Reference Intakes" (RI). Also, in 2013, the United Kingdom introduced the scheme "Traffic Light", and subsequently, in 2017, the "Nutri-Score" label was created by France and shortly, in 2020, "NutrInform Battery" by Italy [7,12,13]. Categorization in FOPL systems has been done in various ways such as in non-interpretive/reductive and interpretive systems. Non-interpretive systems include, for example, the GDA/RIs and the new NutrInform Battery that is according to European Commission (2020) equal to the RI, only with an added battery symbol, while interpretive systems include the Nutri-Score [9,13,14]. Also, another differentiation that is considered important is color labeling. According to Jing Song and his collaborators (2021), color-coded labels gave more impetus to consumers to buy healthier products [15]. It is considered a more understandable and effective method of guiding consumers to healthier food/beverage choices [16].

The impact of each system depends on the consumer's awareness of the meaning of the graphical design [1]. From the research data so far, consumers seem to have a better understanding of FOPL interpretive systems [9] and FOPL color-coded systems that are more effective in guiding consumers [17]. According to the European Commission and the Farm to Fork strategy, a harmonized mandatory food labeling on the front of the package is intended to be proposed in 2022 to help consumers make healthier choices that will benefit their health as well as their overall quality of life while reducing health-related costs [18]. The aim of this study was to investigate consumers' perceptions of the FOPL between two specific labeling systems, the Nutri-Score and the Guideline Daily Amounts (GDA). It could also contribute to the debate on adopting a single evaluation system in the EU, considering the options available.

## Materials and methods

Participants and questionnaire

This is a post hoc analysis of data from a previously published study focusing on specific aspects of perception and subjective understanding of Nutri-Score and Reference Intakes and their sociodemographic determinants [19]. The research methodology is described in detail previously [19]. Participation in the research was voluntary and anonymous. On the first page of the electronic form, the consent of the participants was requested. This study was conducted according to the guidelines laid down in the Declaration of Helsinki, and all procedures involving research study participants were approved by the Ethics Committee of the University of Thessaly (approval report: 24/15.04.2021).

The participants who took part in the research were informed and received the electronic questionnaire from the researchers via personal correspondence, professional correspondence databases, and social media promoted by the method of an avalanche. The research involved Greek people over 18 years old. Two groups were generated. The first group included participants who answered the questionnaire using the GDA label, as the label widely used in packaged products in Greece, and the second group were the participants who answered the questions using the Nutri-Score.

The first part of the questionnaire recorded demographic questions such as gender, age, financial status, and educational level, as well as questions regarding how they judged their diet, what diet they followed, and if they read the nutrition label on the back of the package. The second part of the questionnaire included more specific questions, i.e., a) food selection, b) subjective understanding, and c) objective understanding. Nine perceptions were measured on a five-point Likert scale (1=strongly disagree, 5=strongly agree) [19]. Five questions measured positive perceptions (e.g., “It should be compulsory for this label to be shown on packaged food products,” “I like this label”, “This label is easy to understand”, “This label provides me with the information I need”, and “I trust this label”) and four questions measured negative perceptions (“Food companies should be able to choose whether they apply this label to their packaged foods”, “This label is confusing”, “This label does not stand out”, and “This label took too long to understand”).

Statistical analyses

Data analyses were performed using SPSS Version 23.0 (IBM Corp., Armonk, NY, USA). Normality was tested with the Shapiro-Wilk test by producing histograms, skewness, and kurtosis values indicating that the distribution of perception variables may be considered normal. Independent samples t-tests and one-way analysis of variance (ANOVA) were used to compare the scores of perception variables and demographic and dietary characteristics between groups. To test the effects of the label (GDA vs. Nutri-Score) on overall positive and negative perceptions, a multivariate analysis of variance (MANOVA) was performed. Multivariate and univariate effects were extracted, while the models were adjusted with demographic and dietary variables (gender, age groups, income groups, educational level, healthy diet, proper nutrition, and responsibility for grocery shopping). Positive and negative perceptions scales should present good reliability, i.e., Cronbach’s alpha of 0.805 and 0.629 [20].

## Results

Table 1 presents the characteristics of the study population, including sociodemographic data and nutrition-related characteristics. A total of 510 participants were included in the study, of whom 49.6% were women, 37.1% of the individuals were between 31 and 50 years old, 35.3% declared medium income, 70.8% had a high educational level, 85.7% declared following a healthy diet, 59.8% had a good nutrition knowledge, and 70.2% declared responsible for grocery shopping.

**Table 1:** Demographics and dietary characteristics of the sample (N=510) and comparisons between the two food label groups. GDA: Guideline Daily Amount.

		Food label group				Total	
		GDA		Nutri-Score			
		N	%	N	%	N	%
Gender	Male	128	50.2	129	50.6	257	50.4
	Female	127	49.8	126	49.4	253	49.6
Age groups	18-30	73	28.6	79	31.0	152	29.8
	31-50	99	38.8	90	35.3	189	37.1
	>50	83	32.5	86	33.7	169	33.1
Income groups	Low	101	39.6	70	27.5	171	33.5
	Medium	71	27.8	109	42.7	180	35.3
	High	83	32.5	76	29.8	159	31.2
Following a healthy diet	Yes	220	86.3	217	85.1	437	85.7
	No	35	13.7	38	14.9	73	14.3
Knowing about nutrition	Yes	180	70.6	125	49.0	305	59.8
	No	75	29.4	130	51.0	205	40.2
Responsible for grocery shopping	Yes	179	70.2	180	70.6	359	70.4
	No	76	29.8	75	29.4	151	29.6
Educational level	High	187	73.3	174	68.2	361	70.8
	Medium	68	26.7	81	31.8	149	29.2

Table 2 presents the descriptive statistics of perception scores for each label. Nutri-Score presented higher scores in the item “I like this label” (M=4.20, SD=1.04) compared to GDA (M=3.86, SD=1.02, d=0.328). Moreover, a difference was detected in the item “It should be compulsory for this label to be shown on packaged food products” in favor of the GDA label (M=4.56, SD=0.92) vs. Nutri-Score (M=4.20, SD=1.04, d=0.345). Nutri-Score was also considered easier to be understood (M=4.05, SD=1.12) compared to GDA (M=3.75, SD=1.16, d=0.260). The scores of negative perceptions were in favor of the Nutri-Score. Specifically, participants reported that the GDA label did not stand out (M=2.26, SD=1.21) and took too long to be understood (M=2.33, SD=1.17) in comparison to Nutri-Score (M=1.82, SD=1.12, d=0.363 and M=1.96, SD=1.19, d=0.310, respectively).

**Table 2:** Differences in perceptions between Nutri-Score and GDA labels. GDA: Guideline Daily Amounts.

	Label	Μ	SD	t	p
Food companies should be able to choose whether they apply this label to their packaged foods	GDA	1.95	1.44	-1.203	0.230
	Nutri-Score	2.11	1.43		
This label is confusing	GDA	1.79	1.03	-0.505	0.614
	Nutri-Score	1.84	1.08		
It should be compulsory for this label to be shown on packaged food products	GDA	4.56	0.92	4.096	<0.001
	Nutri-Score	4.20	1.04		
I like this label	GDA	3.86	1.02	-3.751	<0.001
	Nutri-Score	4.20	1.04		
This label does not stand out	GDA	2.26	1.21	4.242	<0.001
	Nutri-Score	1.82	1.12		
This label is easy to understand	GDA	3.75	1.16	-2.999	0.003
	Nutri-Score	4.05	1.12		
This label took too long to understand	GDA	2.33	1.17	3.573	<0.001
	Nutri-Score	1.96	1.19		
This label provides me with the information I need	GDA	3.76	1.09	0.602	0.547
	Nutri-Score	3.70	1.11		
I trust this label	GDA	3.61	1.02	-1.701	0.090
	Nutri-Score	3.76	1.06		

The effect of demographic variables (gender, age, educational level, income) on participants’ perceptions was examined separately for the GDA and Nutri-Score groups. Significant results were extracted only for the Nutri-Score group, as presented in Table 3. Demographics did not affect perceptions in the GDA group. More specifically, men reported to a larger extent compared to a female that Nutri-Score was easy to understand (t(253)=3.66, p<0.001), providing the necessary information (t(253)=3.26, p=0.001), being trustworthy (t(253)=2.30, p=0.002). Participants over the age of 50 years had a more favorable view of the Nutri-Score in terms of how much they liked the label (F(2, 252)=6.50, p=0.002), how easy it is to understand (F(2, 252)=8.88, p<0.001), provision of the necessary information (F(2, 252)=10.25, p<0.001), trust (F(2, 252)=14.90, p<0.001), having a less negative view for the compulsory inclusion of the label in food packages (F(2, 252)=7.42, p=0.001), the label not standing out (F(2, 252)=8.01, p=<0.001), and time taken to understand the label (F(2, 252)=6.48, p=0.002).

**Table 3:** Comparisons between groups regarding the effect of demographics on perceptions of the Nutri-Score label (only statistically significant results are presented). One-way ANOVA statistic (F) for the effect of age groups and income groups, and t-test results statistic (t) for the effect of gender and educational level.

	M	SD	M	SD	M	SD	F/t	p
Gender			Male		Female			
This label is easy to understand			4.30	0.95	3.80	1.22	3.66	<0.001
This label provides me with the information I need			3.92	1.06	3.48	1.13	3.26	0.001
I trust this label			3.91	0.97	3.61	1.13	2.30	0.022
Age groups	18-30		31-50		>50			
Food companies should be able to choose whether they apply this label to their packaged foods	2.46	1.54	2.23	1.44	1.65	1.20	7.42	0.001
I like this label	4.11	1.04	3.98	1.11	4.51	0.88	6.50	0.002
This label does not stand out	1.97	1.07	2.06	1.19	1.44	1.00	8.01	<0.001
This label is easy to understand	3.81	1.14	3.89	1.14	4.45	0.97	8.88	<0.001
This label took too long to understand	2.16	1.17	2.13	1.17	1.59	1.15	6.48	0.002
This label provides me with the information I need	3.39	1.27	3.58	1.06	4.12	0.89	10.25	<0.001
I trust this label	3.47	1.11	3.57	1.01	4.24	0.91	14.90	<0.001
Educational level			Medium		High			
This label does not stand out			1.47	0.90	1.99	1.18	3.51	0.001
This label is easy to understand			4.31	1.06	3.94	1.13	-2.50	0.013
This label took too long to understand			1.60	1.14	2.13	1.18	3.32	0.001
This label provides me with the information I need			3.95	0.99	3.59	1.15	-2.46	0.015
I trust this label			4.00	1.02	3.66	1.06	-2.44	0.015
Income groups	Low		Medium		High			
Food companies should be able to choose whether they apply this label to their packaged foods	2.40	1.64	1.73	1.18	2.37	1.45	6.73	0.001
I like this label	4.23	0.89	4.40	0.95	3.88	1.20	5.94	0.003
This label is easy to understand	3.87	1.15	4.35	1.05	3.80	1.10	6.96	0.001
This label took too long to understand	2.16	1.25	1.73	1.18	2.11	1.10	3.57	0.030
This label provides me with the information I need	3.77	1.07	3.92	1.08	3.33	1.12	6.72	0.001

Less-educated participants presented a more favorable view of the Nutri-Score compared to more-educated participants. The less-educated participants reported to a larger extent that Nutri-Score was easier to understand (t(253)=2.50, p=0.013), providing the necessary information (t(253)=2.46, p=0.015), and was trustworthy (t(253)=2.44, p=0.015). At the same time, less-educated participants had a lower negative perception of the Nutri-Score label in terms of not standing out (t(253)=3.51, p=0.001) and time was needed to understand the label (t(253)=3.32, p=0.001). Participants of medium income presented less frequent negative views for the compulsory inclusion of the label in food packages (F (2, 252)=6.73, p=0.001), time taken to understand the label (F(2, 252)=3.57, p=0.030), more favorable views in terms of liking the label (F(2, 252)=5.94, p=0.003), easiness to understand (F(2, 252)=6.96, p=0.001), time spent to understand (F(2, 252)=3.57, p=0.030), and provision of the necessary information (F(2, 252)=6.72, p=0.001).

Participants' perceptions were investigated according to whether or not they follow a healthy diet and whether or not they are responsible for buying food for the family (Table 4). The participants who followed an unhealthy diet indicated that the GDA label does not stand out (M=2.71, SD=1.34), according to the participants who were following a healthy diet (M=2.19, SD=1.18, t(253)=2.394, p=0.017). Respondents who do not know about nutrition reported that GDA did not stand out (t(253)=2.44, p=0.015), took too long to be understood (t(253)=2.26, p=0.025), and was less easy to be understood (t(253)=2.09, p=0.038), compared to participants with high nutrition knowledge. On the other hand, in the Nutri-Score group, participants with less nutritional knowledge less frequently reported that the label did not stand out (t(253)=2.25, p=0.025), took too long to be understood (t(253)=3.54, p<0.001), and more frequently that the label was easy to be understood (t(253)=2.59, p=0.010), providing the necessary information (t(253)=2.47, p=0.014) being trustworthy (t(253)=2.70, p=0.007), compared to participants with a higher nutrition knowledge.

**Table 4:** Perceptions of the GDA and Nutri-Score labels in terms of dietary variables (only significant results are presented). GDA: Guideline Daily Amounts.

Label		M	SD	M	SD	t	p
Knowledge nutrition		Yes		No			
GDA	This label does not stand out	2.14	1.2	2.55	1.21	-2.44	0.015
	This label is easy to understand	3.85	1.15	3.52	1.16	2.09	0.038
	This label took too long to understand	2.23	1.11	2.59	1.25	-2.26	0.025
Nutri-Score	This label does not stand out	1.98	1.17	1.67	1.06	2.25	0.025
	This label is easy to understand	3.87	1.20	4.23	1.01	-2.59	0.010
	This label took too long to understand	2.22	1.23	1.71	1.10	3.54	<0.001
	This label provides me with the information I need	3.53	1.23	3.87	0.97	-2.47	0.014
	I trust this label	3.58	1.12	3.94	0.98	-2.70	0.007
Responsible for grocery shopping		Yes		No			
GDA	I like this label	3.95	0.99	3.64	1.05	2.21	0.028
	This label took too long to understand	2.22	1.17	2.61	1.11	-2.45	0.015
Nutri-Score	This label is confusing	1.96	1.14	1.56	0.84	2.71	0.007
	I like this label	4.08	1.06	4.49	0.92	-2.96	0.003
	This label does not stand out	1.98	1.20	1.44	0.79	3.60	<0.001
	This label is easy to understand	3.92	1.14	4.39	0.98	-3.11	0.002
	This label took too long to understand	2.12	1.26	1.57	0.90	3.43	0.001
	This label provides me with the information I need	3.57	1.14	4.01	0.99	-2.92	0.004
	I trust this label	3.63	1.09	4.08	0.93	-3.12	0.002

Participants who stated that they were not responsible for purchasing food in the family reported that GDA was preferable (t(253)=2.21, p=0.028) but stated that it took a long time to understand it (t(253) =2.45, p=0.015), compared to participants who were responsible for shopping. On the other hand, in the Nutri-Score group, participants who had no responsibility for purchasing food in the family reported to a lesser extent that the label was confusing (t(253)=2.21, p=0.028), the label did not stand out (t( 253 )=3.60, p<0.001), and it took longer to understand (t(253)=3.43, p=0.001), while we observe that this label was preferred to a greater extent by the participants (t(253)= 2.96, p=0.003) and it was easy to understand (t(253)=3.11, p=0.002), providing the necessary information (t(253)=2.92, p=0.004) and reliability (t( 253)=3.12, p=0.002), compared to participants who were responsible for food shopping.

MANOVA was performed to investigate the multivariate effect of labeling, demographics, and dietary characteristics on the overall scores of positive and negative perceptions, respectively (Table 5). The type of label had a significant moderate effect on positive perceptions (F(5,497)=15.02, p<0.001, η^2^p=0.131) and a significant weak effect on negative perceptions (F(4,498)=7.83, p<0.001, η^2^p=0.059). Moreover, the gender (F(5,497)=3.78, p=0.002, η^2^p=0.037), age group (F(5,497)=2.41, p=0.035, η^2^p=0.024), and following a healthy diet (F(5,497)=2.37, p=0.038, η^2^p=0.023) impacted positive perception. Interaction effects of labels with gender, age groups, and unhealthy diet were examined, and no significant results were found.

**Table 5:** Multivariate effects of label and control variables on positive and negative perceptions. FOPL: front-of-pack label, GDA: Guideline Daily Amounts, df: degrees of freedom.

Variable	Pillai's Trace	F	df	Error df	p	η^2^_p_
Dependent: Positive perceptions						
FOPL (Nutri-Score vs. GDA)	0.131	15.019	5	497	<0.001	0.131
Gender	0.037	3.777	5	497	0.002	0.037
Age groups	0.024	2.413	5	497	0.035	0.024
Income groups	0.021	2.113	5	497	0.063	0.021
Following a healthy diet	0.023	2.370	5	497	0.038	0.023
Having knowledge about nutrition	0.005	0.456	5	497	0.809	0.005
Responsibility for grocery shopping	0.012	1.195	5	497	0.311	0.012
Educational level	0.006	0.637	5	497	0.671	0.006
Dependent: Negative perceptions						
FOPL (Nutri-Score vs. GDA)	0.059	7.831	4	498	<0.001	0.059
Gender	0.014	1.749	4	498	0.138	0.014
Age groups	0.011	1.348	4	498	0.251	0.011
Income groups	0.004	0.445	4	498	0.776	0.004
Following a healthy diet	0.013	1.703	4	498	0.148	0.013
Having knowledge about nutrition	0.003	0.392	4	498	0.814	0.003
Responsibility for grocery shopping	0.008	0.954	4	498	0.432	0.008
Educational level	0.010	1.282	4	498	0.276	0.010

The adjusted univariate effects of labeling on positive perception variables are presented in Table 6. Participants indicated at a larger extent that GDA was compulsory (F(1,501)=17.34, p<0.001, η^2^p=0.033), while Nutri-Score was preferable (F(1,501)=15.41 p<0.001, η^2^p=0.030) and easier to be understood (F(1,501)=8.91, p=0.003, η^2^p=0.017) compared to GDA.

**Table 6:** Univariate effects of labeling on positive perception variables. Results were adjusted to gender, age groups, income groups, following a healthy diet, having knowledge about nutrition, responsibility for grocery shopping, and educational level. CI: confidence interval, GDA: Guideline Daily Amounts, df: degrees of freedom.

			95% CI						
Dependent	Label	Mean	Lower	Upper	df	Error df	F	p	η^2^_p_
It should be compulsory for this label to be shown on packaged food products	GDA	4.565	4.442	4.688	1	501	17.342	<0.001	0.033
	Nutri-Score	4.191	4.068	4.314					
I like this label	GDA	3.847	3.719	3.974	1	501	15.410	<0.001	0.030
	Nutri-Score	4.212	4.085	4.340					
This label is easy to understand	GDA	3.750	3.608	3.891	1	501	8.912	0.003	0.017
	Nutri-Score	4.058	3.917	4.200					
This label provides me with the information I need	GDA	3.759	3.624	3.894	1	501	0.317	0.574	0.001
	Nutri-Score	3.704	3.569	3.839					
I trust this label	GDA	3.615	3.487	3.742	1	501	2.360	0.125	0.005
	Nutri-Score	3.758	3.630	3.886					

Univariate effects of labeling on negative perception variables (Table 7) showed that Nutri-Score was preferable (F(1,501)=18.06, p<0.001, η^2^p=0.035) and easier to be understood (F(1,501)=11.29, p=0.001, η^2^p=0.022) compared to GDA.

**Table 7:** Univariate effects of label on negative perception variables. Results were adjusted to gender, age groups, income groups, following a healthy diet, having knowledge about nutrition, responsibility for grocery shopping and educational level. GDA: Guideline Daily Amounts, df: degrees of freedom.

			95% CI								
Dependent	Label	Mean	Lower	Upper		df	Error df		F	p	η^2^_p_
Food companies should be able to choose whether they apply this label to their packaged foods	GDA	1.95	1.77		2.13	1	501	1.467		0.226	0.003
	Nutri-Score	2.11	1.93		2.29						
This label is confusing	GDA	1.79	1.66		1.92	1	501	0.288		0.592	0.001
	Nutri-Score	1.84	1.71		1.97						
This label does not stand out	GDA	2.27	2.12		2.41	1	501	18.061		<0.001	0.035
	Nutri-Score	1.82	1.68		1.96						
This label took too long to understand	GDA	2.33	2.18		2.47	1	501	11.288		0.001	0.022
	Nutri-Score	1.97	1.82		2.11						

## Discussion

We assessed consumers' perceptions of two labeling systems (GDA vs. Nutri-Score) and found that consumers preferred interpretive labels much better than numerical/reductive labels. The study results highlighted that the Nutri-Score nutrition label was more understandable, clear, and visible than the GDA rating label. These results are consistent with that of Hung et al., who stated that communication should be kept simple and straightforward, yet scientifically sound, as consumers favor health claims with shorter and less complex messages, as well as health symbols with visible endorsement [21]. Furthermore, a meta-analysis performed by Ikonen et al. supported that consumers seem to be positively affected by interpretative summary indicators [22]. Numerous research studies have shown that simple labels reduce the cognitive effort and time required by the consumer to process information compared to more detailed labels or various forms that provide numerical information [23-25].

However, there is a contradiction with the results of the study by Mazzu et al. [14], who investigated the consumer perceptions of the NutrInform Battery label that belongs to the non-interpretive labels compared to the Nutri-Score label. Their results showed that NutrInform Battery was more understandable when informing consumers about the nutrient composition of the product. However, the questions evaluated by Mazzu et al., such as "This label helps me to understand the product composition ", could only be answered positively when participants were exposed to a label with specific nutrients. Therefore, the way the questions are asked also impacts the answers [14].

According to a review by Grunert and Wills, women are more interested in nutrition than men, probably due to weight control and aesthetic concerns, while older people have increased health concerns. In older age groups, this interest may be lost due to difficulties in processing information [26]. In the present study, men stated to a greater extent that the Nutri-Score was easy to understand, providing the necessary information needed to be more reliable in people over the age of 50. According to Bossuyt et al., the presence of Nutri-Score on products can help the elderly make more accurate healthfulness valuations [27]. Also, according to the systematic review conducted by Hersey et al. (2013), consumers can more easily interpret FOP food labels that incorporate text and symbolic color instead of labels that emphasize only numerical information, such as Guidance Daily Quantities, similar to RIs [28].

Another parameter that deserves and should be emphasized is the evaluation of the participants' perceptions according to their educational background. According to the studies conducted, people with a higher level of education could process more complex dietary statements and were more likely to have healthier eating habits [29,30]. In the present study, we found that the less-educated participants reported to a greater extent that the Nutri-Score was easy to understand, providing the necessary information that needed to be more reliable. In a recent study conducted in Great Britain, people with a lower level of education showed better perception results in some products labeled with Nutri-Score [31]. Numerous studies comparing different FOPL food labels showed that the Nutri-Score is the easiest, most understandable, and considered the most preferred label among many consumers [32]. In general, simpler FOP labels are more effective in helping people with lower education to find healthier products [33].

Age, social background, interest in healthy eating, and nutrition knowledge seemed to play an essential role in understanding nutritional information [34]. We found that respondents who said they do not have much nutritional knowledge reported that GDA did not stand out and it took a long time to understand compared to participants with better nutrition knowledge. In contrast to the Nutri-Score group, participants with less nutritional knowledge reported to a greater extent that the label was easy to understand, providing the necessary information being reliable compared to participants who knew about nutrition. Our results are consistent with other studies showing that people with poor nutritional knowledge had more difficulty understanding detailed FOP labels [35].

According to Melissa Burton and her colleagues (2017) study, a new term "food literacy" was introduced in 2016, including the knowledge, skills, and behaviors required for daily nutrition [36]. In this direction, we evaluated how people who declared themselves responsible for grocery shopping for the family perceived and comprehended the two FOPLs (GDA and Nutri-Score). Sah et al. showed that responsibility promotes healthy choices, while enjoyment prevents them, especially within the family [37]. Our results showed that those responsible for grocery shopping and those who were not responsible for grocery shopping liked the Nutri-Score label more than the GDA label. 

The same results were presented in the study by Talati et al. [38], where the participants reported that the GDA label did not stand out and was confusing, although it should be mandatory on the packages. According to Bandeira et al. and Deliza et al., it should be emphasized that familiar labels can improve efficiency [39,40].

According to Dereń and colleagues, the best choice for labels on the front of food packaging is color labels that are easy to understand and quickly interpreted by all consumers promoting health regardless of socioeconomic background. Nutri-Score meets these criteria and has proven to be an effective labeling tool [11]. The use of color coding with the multicolor scale (green-red) is internationally understood [41].

In the present study, some limitations should be mentioned. A significant limitation was that she created a convenient sample over-representing the highly educated population. However, there was sufficient variability within the sample, permitting evaluation of the effects of various socioeconomic determinants. Also, a large percentage of participants self-reported that they followed a healthy diet and considered that they had enough nutrition knowledge, probably due to the high percentage of people with a high level of education.

## Conclusions

The present study's findings on Greek consumers showed that the interpretive Nutri-Score label was superior to the non-interpretive/numerical GDA because the Nutri-Score (FOPL) was more understandable, clear, and visible to the participants than the GDA (FOPL). In addition, we found that the less-educated participants and men above 50 years old reported to a greater extent that the Nutri-Score was easy to understand, providing the necessary information. Interpretative front-of-pack labels (FOPLs) can improve the ability of consumers in Greece to understand the quality of food products. Public awareness campaigns, in the context of public health, are considered necessary for understanding nutritional labeling (FOPL) and consequently improving dietary choices.
